# *Notes from the Field:* Measles Outbreak on an Army Post and a Neighboring Community — El Paso, Texas, July–September 2019

**DOI:** 10.15585/mmwr.mm6923a2

**Published:** 2020-06-12

**Authors:** Joshua Vance, Fernando Gonzalez, Elizabeth Estrada, Hector I. Ocaranza, Nakia Clemmons, Vanessa Palacios

**Affiliations:** ^1^Public Health Region 9/10, Texas Department of State Health Services; ^2^City of El Paso Department of Public Health, Texas; ^3^United States Army Public Health Nursing, Fort Bliss, Texas; ^4^National Center for Immunization and Respiratory Diseases, CDC.

On July 3, 2019, Army Public Health (APH), located at Fort Bliss, Texas, received a report of a suspected case of measles in a woman who worked at Fort Bliss. The woman did not live on the post and had no recent reported travel. Fort Bliss, one of the largest U.S. Army posts, is located in El Paso County, Texas, which has >800,000 residents* and shares a border with Mexico and the city of Juarez, with a population of 1.4 million.^†^ The last confirmed measles case reported in El Paso County, Texas, was in 1993, and the last outbreak occurred in 1990 (*1*). The same day, the City of El Paso Department of Public Health (CEPDPH) alerted the Texas Department of State Health Services (TDSHS) of another suspected measles case in an unvaccinated El Paso County resident, aged 3 years, who lived on Fort Bliss, also had no recent travel, and whose father was an active-duty soldier. On July 9, both cases were confirmed by reverse transcription–polymerase chain reaction testing at the TDSHS laboratory in Austin.

CEPDPH immediately issued advisories to local medical providers and began contact tracing of confirmed cases. Preexisting immunization clinics extended their hours to provide measles, mumps, and rubella (MMR) vaccine, and immune globulin was requested from TDSHS for postexposure prophylaxis for infants, pregnant women, and immunocompromised persons. TDSHS initiated daily telephone calls with CEPDPH and APH to coordinate prevention and control efforts. CEPDPH established a telephone help line to field concerns among community members, deployed an education task force throughout the county, sent letters to all local school district superintendents, and actively communicated with Mexican health officials located directly across the U.S. border. At Fort Bliss, use of military child care facilities and youth service programs were restricted to children who were up to date with MMR vaccinations^§^ (*2*). APH actively monitored all Fort Bliss medical facilities for new cases and held daily meetings with Fort Bliss senior leaders. TDSHS monitored a statewide syndromic surveillance system to identify persons with measles-like symptoms. At the end of July, a CDC team was invited to El Paso to provide epidemiologic support.

Four additional cases were confirmed at the TDSHS laboratory in Austin, bringing the total number of cases to six; all rash onset dates occurred during June 30–July 19. Fort Bliss–associated cases included one in a child and two in adults, neither of whom were active duty personnel. Among the six cases, three cases occurred in children aged 1–4 years, all of whom were completely unvaccinated (i.e., had not received MMR or any other vaccines); the other three were in adults. One adult patient had laboratory evidence of immunity suggesting previous vaccination; vaccination status of the other two adult patients was unknown. Genotyping by CDC and the Minnesota Vaccine Preventable Disease Reference Center revealed an identical measles strain (D8) in all six patients. A total of 91 specimens from patients with measles-compatible symptoms were tested at the TDSHS laboratory during July 3–September 3; several specimens were also tested for rubella, but no cases of rubella were diagnosed.

Interviews with all six patients or their proxies found that, approximately 2 weeks before their rash onsets, the first two patients visited the same large shopping center where it is possible that exposure to a person with undiagnosed measles could have occurred. Despite investigation into how the first two patients were infected, the primary case for this outbreak remains unidentified. Similarly, interviews with the four other patients or their proxies failed to identify any epidemiologic links. On September 3, 2019, the outbreak was declared over, after two incubation periods (total of 42 days) without occurrence of a new case.

Measles remains a risk to unvaccinated persons in the United States. Thus, although the coordinated prevention and control measures implemented by CEPDPH, APH, TDSHS, and CDC likely prevented a larger outbreak, this event served as an important reminder that persons without presumptive evidence of immunity to measles, mumps, and rubella^¶^ should receive MMR vaccine according to published recommendations by the Advisory Committee on Immunization Practices (*2*).

## Footnotes

* Includes service members stationed at Fort Bliss and their families. http://www.census.gov/quickfacts/fact/table/elpasocountytexas#.

† http://data.un.org/Data.aspx?q=mexico+city&d=POP&f=tableCode%3A240%3BcountryCode%3A484.

§ Children who had received all MMR vaccine doses recommended by the Advisory Committee on Immunization Practices appropriate for their current age.

¶ Documentation of receipt of ≥1 dose of measles-containing vaccine on or after the first birthday for preschool-aged children and adults not at high risk for exposure or 2 doses for school-aged children and adults at high risk; or laboratory evidence of immunity; or laboratory confirmation of disease; or birth before 1957.
